# Fbxo7 and Pink1 play a reciprocal role in regulating their protein levels

**DOI:** 10.18632/aging.202236

**Published:** 2020-12-03

**Authors:** Tianwen Huang, Lijun Fang, Raoli He, Huidan Weng, Xiaochun Chen, Qinyong Ye, Dianbo Qu

**Affiliations:** 1 Department of Neurology, Fujian Medical University Union Hospital, Fuzhou, China; 2 Department of Ophthalmology, Fujian Medical University Union Hospital, Fuzhou, China; 3 Hotchkiss Brain Institute, Department of Clinical Neurosciences, University of Calgary, Calgary T2N 4N1, Alberta, Canada

**Keywords:** Parkinson's disease, Fbxo7, Pink1

## Abstract

Pink1, Parkin and Fbxo7, three autosomal recessive familial genes of Parkinson’s disease (PD), have been implicated in mitophagy pathways for quality control and clearance of damaged mitochondria, but the interplay of these three genes still remains unclear. Here we present that Fbxo7 and Pink1 play a reciprocal role in the regulation of their protein levels. Regardless of the genotypes of Fbxo7, the wild type and the PD familial mutants of Fbxo7 stabilize the processed form of Pink1, supporting the prior study that none of the PD familial mutations in Fbxo7 have an effect on the interaction with Pink1. On the other hand, the interaction of Fbxo7 with Bag2 further facilitates its capability to stabilize Pink1. Intriguingly, the stabilization of Fbxo7 by Pink1 is specifically observed in substantial nigra pars compacta but striatum and cerebral cortex. Taken together, our findings support the notion that Fbxo7 as a scaffold protein has a chaperon activity in the stabilization of proteins.

## INTRODUCTION

Parkinson’s disease (PD) is the second most common progressive neurodegenerative disease after Alzheimer’s disease, characterized by tremor, rigidity and bradykinesia due to dopaminergic (DA) loss in substantial nigra pars compacta (SNc) [1, 2]. It is widely accepted that the environmental and genetic factors play a crucial role together in the pathogenesis of PD [3]. The growing body of evidence indicates that at least 10% of PD cases are associated with genetic alterations, and up to 30% of PD cases are linked to genetic risk factors [4–6]. To date, the mutations in at least 13 genes have been linked to the familial form of PD [7]. Of those, the mutations in Fbxo7 (F-box domain containing protein 7; PARK15) cause an autosomal recessive early onset PD [8, 9]. The symptoms caused by Fbxo7 mutations are similar to those caused by Pink1 (PTEN-induced putative kinase 1; PARK6) or Parkin (RBR E3 ubiquitin-protein ligase; PARK2) mutations [8–10], suggesting that the crosstalk of these three genes plays an essential role in the pathogenesis of PD. In fact, Fbxo7 and Parkin act in the same signaling pathway to regulate mitophagy [11]. None of the familial mutations of Fbxo7 functionally rescues the phenotype induced by the Parkin mutant in drosophila and the impaired mitophagy mediated by Parkin in cells, while the wild type (WT) Fbxo7 does [11]. However, it is noteworthy to point out that the familial mutation R378G of Fbxo7 rather than others can rescue Parkin translocation during mitophagy [11]. In addition, neither the WT Fbxo7 nor the PD familial mutants of Fbxo7 functionally rescues the phenotype caused by Pink1 deficiency in drosophila [11]. This data indicates that the interplay between Fbxo7 and Parkin relies not only on facilitating Parkin translocation but also on regulating Parkin mediated subsequent process of mitophagy.

Pink1, a mitochondrially localized serine/threonine kinase gene, acts as an important upstream regulator of Parkin in control of mitophagy [12–18]. As a sensor protein, Pink1 rapidly responds to mitochondrial stressors such as CCCP to recruit Parkin to mitochondria, where Parkin will be activated to facilitate mitophagy [13–18]. Although the overexpression of Fbxo7 is unable to rescue the phenotypes of Drosophila with an expression of the Pink1 mutant, Pink1 can accentuate Fbxo7 translocation to mitochondria [11]. Taken together, the data indicates that the activity of Pink1 kinase probably plays a regulatory role in the Fbxo7-mediated mitophagy.

Pink1 is processed by multiple proteases in the inner mitochondrial membrane [19–21]. The processed form of Pink1 (PF-Pink1) is then transported to the cytoplasm for degradation through a proteasomal pathway [22, 23], where the 52-kD form of Pink1 is ubiquitinated by a chaperon dependent E3 ligase CHIP and degraded by proteasome machinery [24, 25]. However, this process of Pink1 proteasomal degradation is also able to be regulated by Bag (Bcl-2 associated athanogene) family proteins. For example, Bag2 and Bag5 play an inhibitory role in the proteasomal degradation of Pink1 [26–28], whereas Bag6 acts oppositely [29]. Together, these lines of evidence indicate that both ubiquitin ligases and chaperon proteins play unignorable roles in Pink1 stability. Given that F-Box containing proteins are essential for recognition of target proteins to be ubiquitinated [30–32], we sought to examine in detail whether Fbxo7 could impact the stability of Pink1 or vice versa. Our findings demonstrate that both Fbxo7 and its PD familial mutants, as a scaffold protein, together with Bag2, probably stabilize Pink1 with their chaperon activity.

## RESULTS

### Fbxo7 and its PD familial mutants stabilize PF-Pink1

**Figure 1:**
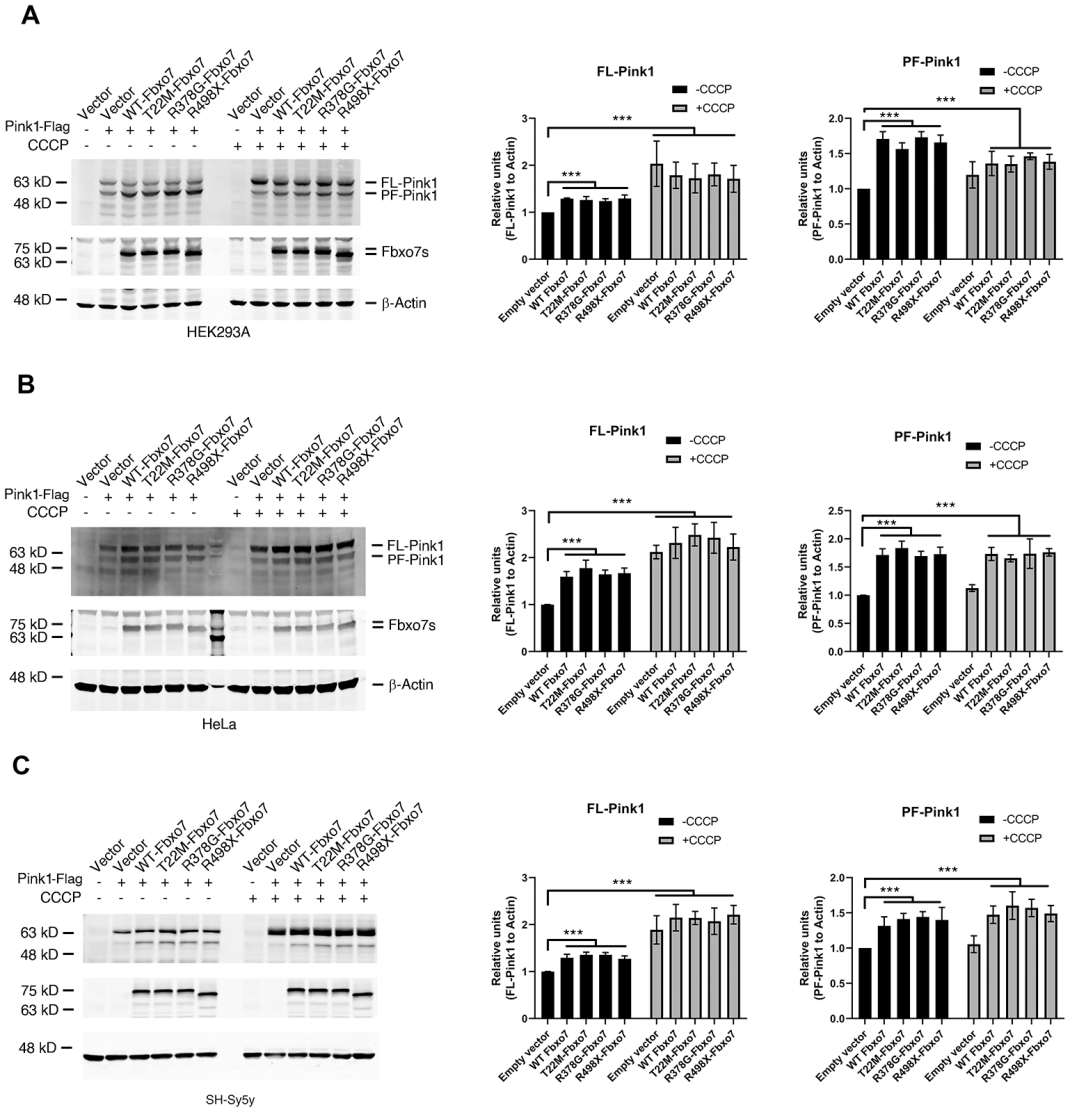
**Expression of Fbxo7 and its PD familial mutants led to an accumulation of Pink1.** The Pink1-Flag plasmid alongside Fbxo7-Myc or its mutated plasmids were co-transfected to three different cell lines (**A**: HEK293A, **B**: HeLa and **C**: SH-Sy5y). 24 hours after transfection, the cells were treated by 10 μM CCCP for 2 hours. The total cell lysates were subjected to Western blot analyses by anti-Flag antibody for Pink1-Flag, anti-Myc antibody for Fbxo7-Myc, and anti-β-Actin for β-Actin. After the relative level of FL-Pink1 and PF-Pink1 was obtained by normalizing of two forms of Pink1 to β-Actin respectively, the relative ratio of the two forms of Pink1 was obtained by normalization of the relative level of the two forms of Pink1 to the control only transfected with Pink1. The relative ratios were shown as mean ± SD; n = 3 independent experiments; individual 2-way ANOVAs with Tukey’s multiple comparisons test; ***p<0.001.

Although previous study demonstrated that Fbxo7 and its PD familial mutants directly interact with Pink1 [11], the effect of Fbxo7 and its PD familial mutants on Pink1 level has yet been clearly elucidated. To test whether Fbxo7 and its PD familial mutants influence the Pink1 level, we overexpressed Fbxo7 and its PD familial mutants along with Pink1 in three different human cell lines, HEK 293A, HeLa and SH-SY5Y. We detected accumulations of PF-Pink1 protein of approximately 52 kD in all three cell lines expressing Fbxo7 or its PD familial mutants when compared to the control cells expressing an empty plasmid by Western blot analyses (Figure 1), regardless of whether the cells were treated with carbonyl cyanide 3 chlorophenylhydrazone (CCCP) or not. Noticeably, upon CCCP treatment, an indistinguishable accumulation of the full-length form of Pink1 (FL-Pink1) from all experimental samples was observed (Figure 1). Collectively, our data indicates that the overexpression of Fbxo7 and its PD familial mutants can stabilize PF-Pink1 in different human cell lines.

### Tuning the protein levels of Fbxo7 alters endogenous Pink1 level

**Figure 2:**
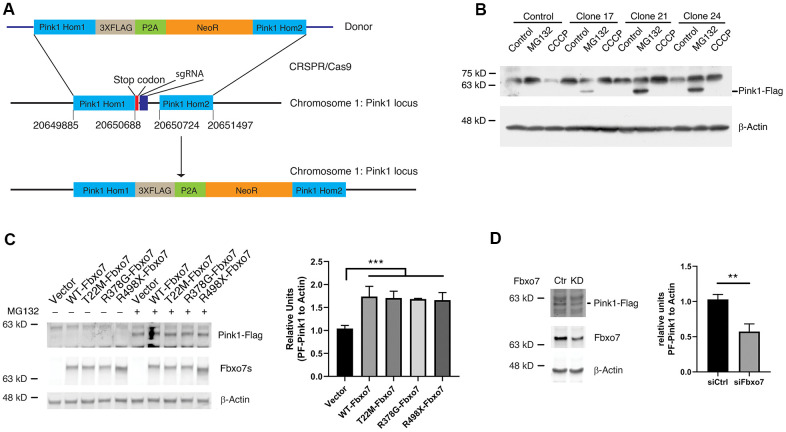
**Expression of Fbxo7 and its PD familial mutants resulted in an accumulation of endogenous Pink1 in a Pink1-Flag KI cell line.** (**A**) a schematic illustration of an engineering strategy for generation of a Pink1-Flag KI cell line. Pink1 Hom1: Pink1 homology arm 1; NeoR: neomycin resistant gene; Pink1 Hom2: Pink1 homology arm 2. (**B**) confirmation of expression of Pink1-Flag in the positive Pink1-Flag KI clones with an anti-Flag antibody. The cells were treated by CCCP and MG-132. (**C**) expression of Fbxo7 and its PD familial forms led to accumulation of PF-Pink1. The plasmids harboring Fbxo7 or the PD associated mutations in Fbxo7 were transfected into the Pink1-Flag KI cells. The total cell lysates were analyzed by Western blots with anti-Flag and anti-Myc antibodies. After the relative level of Pink1 protein was obtained by normalizing of Pink to β-Actin, the relative ratio of Pink1 was obtained by normalization of the relative level of Pink1 from Fbxo7 transfected samples to the control only transfected with Pink1. The relative ratios were shown as mean ± SD; n = 3 independent experiments; individual 2-way ANOVAs with Tukey’s multiple comparisons test; ***p = 0.0007. (**D**) KD of Fbxo7 caused a decrease of PF-Pink1. The Fbxo7 siRNA and control siRNA were transfected into the Pink1-Flag KI cells. 14 hours after transfection, the cells were treated by 10 μM MG-132 for 2 hours. The total cell lysates were analyzed by Western blots with anti-Flag and anti-Fbxo7 antibodies. After the relative level of Pink1 protein was obtained by normalizing to β-Actin, the relative ratio of Pink1 was obtained by normalization of the relative level of Pink1 from siFbxo7 transfected samples to the control transfected with siCtrl. The relative ratios were shown as mean ± SD; n = 3 independent experiments; unpaired 2-tailed Student’s *t* test; **p = 0.0058.

Due to the lack of specificity and efficiency of commercialized Pink1 antibodies to detect the endogenous Pink1, we decided to create a knockin (KI) HEK 293A cell line, in which a cassette containing 3XFlag, a P2A motif and a neomycin resistant gene was integrated into the Pink1 locus in chromosome 1 for the expression of Pink1 fused with 3XFlag at the c-terminus of Pink1and a cleaved form of neomycin driven by P2A. To do this, we constructed a donor plasmid carrying a cassette with 3XFlag, P2A, neomycin, and two arms homologous to target the regions of the Pink1 locus on Chromosome 1, and a plasmid carrying genes for CRSPR/Cas9, tracer RNA and sgRNA specific for creating a break near the stop codon of Pink1 (Figure 2A). 24 hours after co-transfection of the donor and the sgRNA plasmids into HEK 293A cells, 0.5 mM G148 was used to select the cells in which the homologous recombination took placed and the cassette containing 3XFlag-P2A-neomycin was integrated into the Pink1 locus. The colonies developed from an individual cell were screened by PCR and the positive clones were further confirmed by western blots using an anti-Flag antibody. PF-Pink1 was observed upon MG132 treatment (Figure 2B). Due to the size of a nonspecific band similar to FL-Pink1, the two were indistinguishable from one another (Figure 2B). Given that the anti-Flag antibody is able to recognize PF-Pink1 from the Pink1-KI cells, we employed the Pink1-KI cell line to examine whether manipulating Fbxo7 levels alters the protein level of Pink1. Consistent with the data from the co-expression of Fbxo7 and Pink1, the increased level of endogenous Pink1 was detected in the Pink1 KI cells expressing Fbxo7 and its PD familial mutants (Figure 2C) when compared to the empty vector transfection control. In contrast, the decreased level of endogenous Pink1 was observed upon the knockdown (KD) of Fbxo7 by siRNA (Figure 2D).

### Fbxo7 and its PD familial mutants has no effect on ubiquitination of Pink1

**Figure 3:**
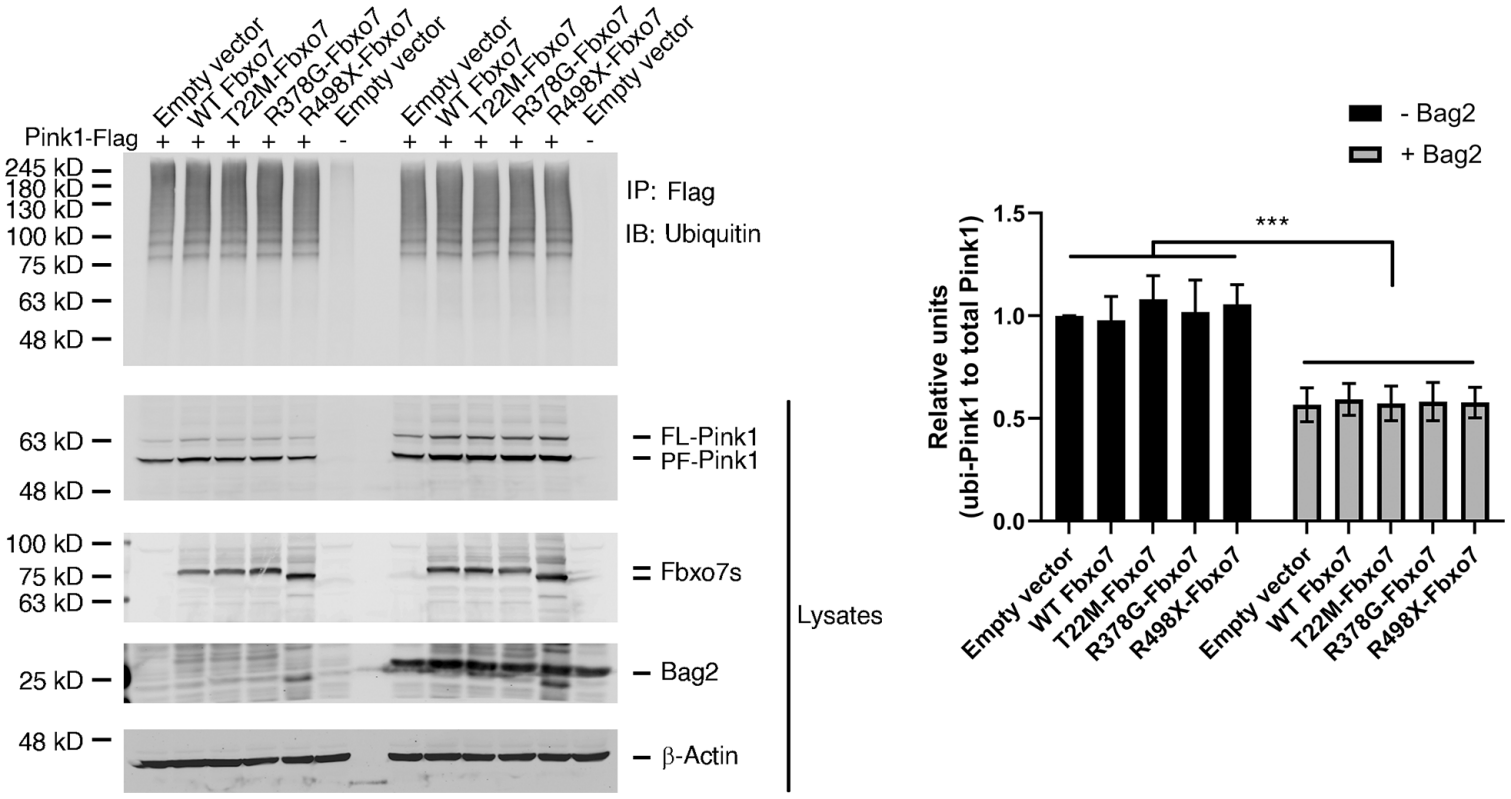
**Expression of Fbxo7 and its PD familial mutants had no effect on Pink1 ubiquitination.** The Pink1-Flag plasmid along with Fbxo7-Myc or its mutated plasmids were co-transfected into HEK 293A cells. 24 hours after transfection, the cells were treated by 10 μM MG132 for 2 hours. The total cell lysates were subjected to immune-precipitation (IP) with anti-Flag antibody after 5-minute boiling. The IPed samples and the total cell lysates were analyzed by western blot with anti-ubiquitin antibody for ubiquitinated Pink1, anti-Flag antibody for Pink1-Flag, anti-Myc antibody for Fbxo7 and Bag2. The total cell lysates were analyzed by Western blots with anti-Flag and anti-Myc antibodies. After the relative level of ubiquitinated Pink1 protein was obtained by normalizing of ubiquitinated Pink to total Pink1, the relative ratio of ubiquitinated Pink1 was obtained by normalization of the relative level of ubiquitinated Pink1 from Fbxo7 or Bag2 transfected samples to the control transfected with Pink1 only (top panel from IP). The relative ratios were shown as mean ± SD; n = 3 independent experiments; individual 2-way ANOVAs with Tukey’s multiple comparisons test; ***p<0.001.

Fbxo7, as a subunit of SCF E3 ubiquitin ligase, is involved in the regulation of proteasomal activity by recruiting its substrates [33]. To test whether Fbxo7 regulates the ubiquitination of Pink1, Fbxo7 and its PD familial mutants along with Pink1 were transfected into HEK 293A. As expected, Pink1 displayed a smear pattern in the presence of Fbxo7 or its PD familial mutants (Figure 3). Surprisingly, there was no difference of the ubiquitinylated level of Pink1 in all samples (Figure 3), suggesting that the proteasomal activity of Fbxo7 may play a negligible role in the stabilization of PF-Pink1. Previously, we reported that the expression of Bag2 inhibits the level of ubiquitylated Pink1 to implement Pink1 stabilization [26]. To dissect whether Bag2 has a synergic role in Fbxo7-mediated accumulation of Pink1, we co-expressed Bag2 and Pink1 along with Fbxo7 or its familial mutants of PD in the HEK 293A cells. We found that Bag2 dramatically reduced the level of ubiquitinylated Pink1 (Figure 3) and further stabilized PF-Pink1 in synergy with the expression of Fbxo7 or the PD familial forms of Fbxo7.

### Fbxo7 directly interacts with Bag2 but Bag5

**Figure 4:**
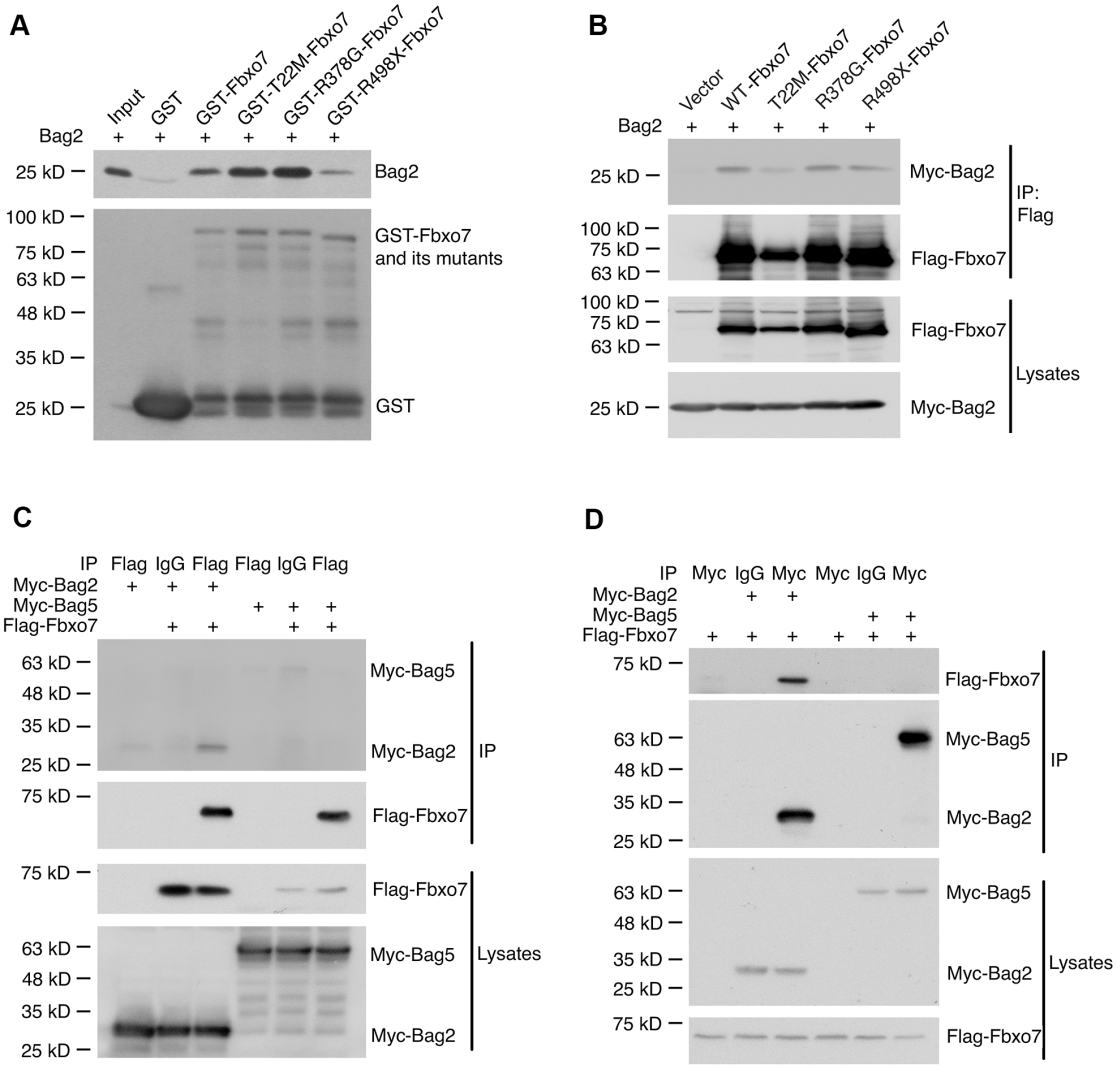
**Specific interaction of Bag2 with Fbxo7.** (**A**) the bacterially expressed GST-Fbxo7 or its mutants was co-incubated with the bacterially expressed His-Bag2 for in vitro binding assay. The pulled-down proteins were analyzed by western blot using anti-Bag2 antibody for recombinant His-Bag2 and anti-GST antibody for GST and GST-Fbxo7. (**B**), the plasmids carrying Fbxo7 or its mutants were co-transfected with a plasmid expressing Bag2 into HEK293A cells. Fbxo7 proteins were IPed by anti-Flag antibody. The IPed proteins were subjected to western blotting. (**C,** **D**) the plasmid expressing Fbxo7 were transfected with the plasmid expressing Bag2 or Bag5 into HEK 293A. The target proteins were IPed by anti-Flag or anti-Myc antibody and the IPed proteins were detected by western blot.

We have demonstrated that Bag2 is capable of inhibiting the ubiquitination of Pink1 and stabilizing the endogenous PF-Pink1. We then asked whether Bag2 directly interacts with Fbxo7 and the PD familial forms of Fbxo7. To this end, we carried out an in vitro binding assay using the recombinant proteins purified from E. coli. The in vitro binding assay revealed that Bag2 directly interacted with Fbxo7 and the PD familial forms of Fbxo7 (Figure 4A). Likewise, we also confirmed the interaction of Bag2 and Fbxo7 or its PD familial mutants in the heterologous HEK 293A cells (Figure 4B). To further confirm the specificity of the interaction between Fbxo7 and Bag2, we performed reciprocal co-immunoprecipitation (IP) analyses in the heterologous cells. We found that Fbxo7 specifically interacted with Bag2 but not Bag5 (Figure 4C, 4D), implicating that the specific interaction between Fbxo7 and Bag2 may have a synergic role in the stabilization of PF-Pink1. Indeed, an increased accumulation of PF-Pink1 was detected from the cells with an overexpression of Bag2 along with Fbxo7 or its PD familial mutants when compared to the control cells with only an overexpression of Fbxo7 or its PD familial mutants (Pink1 western blot for total cell lysates in Figure 3).

### Pink1 reversely affects the protein level of Fbxo7

**Figure 5:**
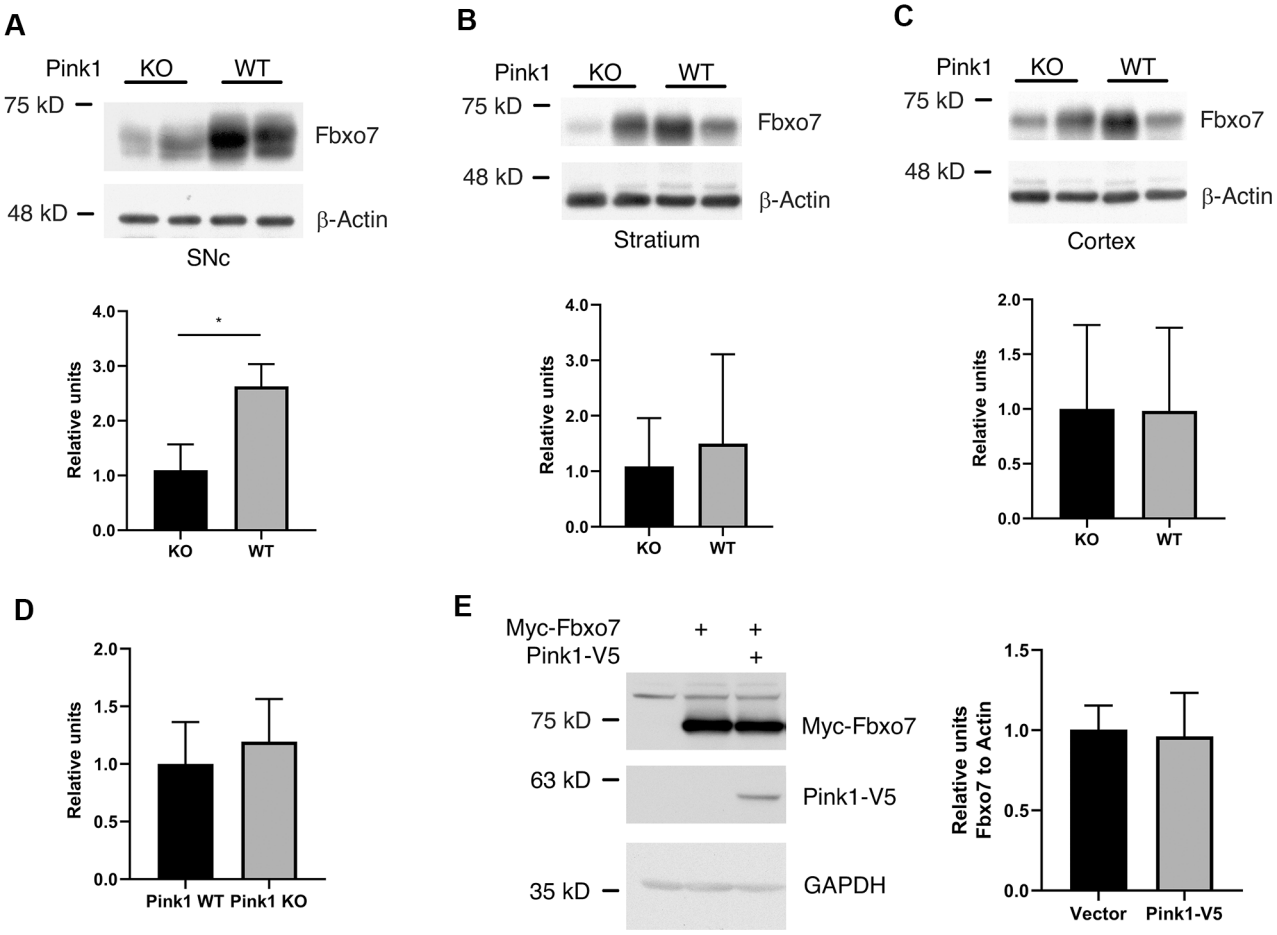
**Pink1 stabilized Fbxo7 in SNc of mouse brain.** The different parts of brain tissues were dissected from WT and Pink1 KO female mice with age of 13-16 weeks. The homogenates were analyzed by western blot to check the protein level of Fbxo7 (**A**–**C**: The protein level of Fbxo7 was normalized to β-Actin, then the relative level of Fbxo7 from WT was further normalized to that from KO). The relative ratios were shown as mean ± SD; n = 4 independent experiments; unpaired 2-tailed Student’s *t* test; **p = 0.0028 for SNc; p = 0.5588 for Stratium; p = 0.9763 for Cortex. (**D**) the mRNAs of Fbxo7 from SNc of WT and KO were analyzed by RT-qPCR. The relative ratios were shown as mean ± SD; n ≥ 5 independent experiments; unpaired 2-tailed Student’s *t* test; p = 0.392. (**E**) expression of Pink1 had no effect on Fbxo7 in HEK 293A cells. The relative ratios were shown as mean ± SD; n = 3 independent experiments; unpaired 2-tailed Student’s *t* test; p = 0.8255.

Burchell et. al. reported that the expression of Fbxo7 is unable to rescue the phenotype caused by Pink1 mutant in a Drosophila model of PD and pinpointed that Pink1 kinase activity is inevitable for functional regulation of Fbxo7 in Pink1-Parkin signaling cascade [11]. We then asked whether Pink1 alters the protein level of Fbxo7. In order to examine the regulatory effect of Pink1 on Fbxo7, the protein extracts from SNc, Striatum and Cortex of WT or KO Pink1 mice were subjected to western blotting analyses. Strikingly, we only observed a decreased change of Fbxo7 in SNc but not in other tissues from the Pink1 KO mice, indicating that the regulation of Pink1 on Fbxo7 may be tissue- or cell-specific (Figure 5A–5C). To determine whether the change of Fbxo7 is transcriptionally regulated in SNc, the level of Fbxo7 mRNA was evaluated by reverse-transcription quantitative PCR (RT-qPCR). The RT-qPCR result unveiled no significant difference of Fbxo7 mRNA from WT and Pink1 knockout SNc (Figure 5D). On the other hand, we examined whether the expression of Pink1 alters Fbxo7 level in cells. To this end, Pink1 and Fbxo7 were co-expressed in HEK 293A cells for western blot analysis. As shown in Figure 5E, the expression of Pink1 had no effect on Fbxo7 level. It least supports the observation from the tissues that a correlated decrease of Fbxo7 is cell specific in Pink1 KO mice.

## DISCUSSION

The work of the past few years has documented that mutations in Fbxo7 are associated with a severe form of autosomal recessive PD [8, 9, 34]. In terms of the molecular functions of Fbxo7, it attributes to mitophagy for the clearance of damaged mitochondria through the interplay with Pink1 and Parkin [11, 35], and to proteasomal pathway for the removal of the damaged, misfolded or unneeded proteins [31, 33, 36]. Importantly, the mice with conditional Fbxo7 KO develop the phenotypes which recapitulate the features of PD including dopaminergic loss and motor dysfunctions [31, 37]. However, the molecular function of Fbxo7 in PD still remains unclear.

The growing lines of evidence demonstrate that Fbxo7 is a subunit of the SCF E3 ligase that promotes proteasomal activity via facilitating the recognition of substrates [31, 33, 36]. Given that Pink1 directly interacts with Fbxo7 and its PD familial mutants [11], it is noteworthy to assess the effect of Fbxo7 and its PD familial mutants on the ubiquitination of Pink1. Accordingly, we evaluated whether the expression of Fbxo7 and its PD familial mutants affects the ubiquitination of Pink1 in three cell lines. We did not observe an increase of ubiquitination level of Pink1 in all samples with the expression of Fbxo7 or its PD familial mutants, regardless of Fbxo7 genotypes. The ubiquitination assay data somehow indicated that Fbxo7 and its PD familial mutants have no effect on the ubiquitination of Pink1. Controversially and in contrast, we observed an unexpected role of Fbxo7 and its PD familial mutants in the stabilization of PF-Pink1, which does not support our original hypothesis that the expression of, at least, WT Fbxo7 should reduce the level of the Pink1 protein. Given that CHIP E3 ligase ubiquitinates PF-Pink to facilitate Pink1 degradation through proteasomal pathway [24], the direct interaction of Fbxo7 and Pink1 may interfere with the recognition of CHIP E3 Ligase to Pink1, which therefore impedes the proteasomal degradation of Pink1. As a matter of fact, it has been reported that Fbxo7 and its PD familial mutants can stabilize Gsk3β and Tom20 [38], and Fbxo7, as a scaffold protein, can stabilize cell cycle regulators, Cdk6, p21 and p27 [39]. In agreement with these lines of evidence, our findings also support the notion that Fbxo7 probably has a feature as a scaffold with chaperon activity. Liu et. al. recently reported that Fbxo7 can ubiquitinate Pink1 in an in vitro ubiquitination assay using purified proteins and KD or expression of Fbxo7 results accumulation or decrease of Pink1 in BEAS-B2 cell line [40]. However, their findings are in disagreement with our results. The reasonable explanations are probably due to first the cell-type difference and second the in vitro system that they used for ubiquitination assay. Nonetheless, in order to elucidate the cell-specific ubiquitination of Pink1mediated by Fbxo7, a variety of cells and tissues should be further explored.

Previously, it has been reported that Bag2 has a critical role in the stabilization of Pink1 through inhibiting the ubiquitination of Pink1 [26, 27]. In line with the previous study, we found that Bag2 can inhibit the ubiquitination of Pink1 to stabilize PF-Pink1 in the cells with the expression of Fbxo7. Given Bag2 has an inhibitory activity on CHIP E3 ligase and a cochaperone activity on stabilization of proteins [24, 26], Bag2 mediated the decrease of Pink1 ubiquitination and the accumulation of PF-Pink1are likely due to first, the inhibition of CHIP E3 ligase activity by Bag2, and second, co-chaperone activity of Bag2 to facilitate Fbxo7 in stabilization of PF-Pink1. Taken together, the data suggests that the inhibition of Pink1 proteasomal degradation is dependent on the synergic chaperon activity of Bag2 and Fbxo7 through their direct interaction.

In addition to the effect of Fbxo7 on Pink1, we found that the loss of Pink1 causes a decrease of Fbxo7 in SNc rather than other brain tissues such as striatum and cortex, raising a question as to whether the expression of Pink1 has a positive effect on the protein level of Fbxo7. Burchell et. al. reported that the expression of Fbxo7 fails to rescue the phenotype caused by Pink1 mutant in drosophila and suggested the importance of Pink1 kinase activity in Fbxo7 mediated mitophagy [11]. To extend this mechanism to our study, it is likely that the Pink1 kinase activity plays a role in regulating the protein level of Fbxo7. This raises a possibility that Fbxo7 might be a substrate of Pink1. To this end, it is of importance to investigate whether Pink1 directly phosphorylates Fbxo7, subsequently modulates the biological function of Fbxo7 in the future study. Given that the PD associated symptoms are absent in Pink1 KO mouse but present in the Pink1 KO rat [41, 42], it will be feasible to exploit the reciprocally functional roles of Pink1 and Fbxo7 in the Pink1 KO rat. In summary, our study highlights the crosstalk of Fbxo7 and Pink1 in regulating their protein levels, and Fbxo7 having a complicated role in the Pink1-Parkin signaling cascade in different types of tissue or cell.

## MATERIALS AND METHODS

### Mice

Pink1 KO mice (C57BL/6J-Pink1^em1Smoc^, NM-KO-191011) were purchased from Shanghai Model Organisms, China. All animal procedures were approved by the Institutional Animal Care and Use Committee of Fujian Medical University. Animals were maintained in strict accordance with the Guidelines for the Use and Treatment of Animals put forth by the NIH’s Guidelines for the care and use of Laboratory Animals.

### Antibodies

Mouse anti-Fbxo7 (Santa Cruz, sc-271763), mouse anti-GAPDH (Santa Cruz, sc-365062), mouse anti-Flag (Sigma, F1804), mouse anti-β-Actin (Sigma, A2228), mouse anti-Myc (Santa Cruz, sc-40), mouse anti-V5 (Bio-Rad, MCA1360GA), mouse anti-Ubiquitin (Abcam, ab134953), rabbit anti-Bag2 (Abcam, ab79406), mouse anti-HA (Abcam, ab130275), anti-mouse horseradish peroxidase-conjugated secondary antibody (Rockland, 18-8817-31) and anti-rabbit horseradish peroxidase-conjugated secondary antibody (Rockland, 18-8816-31) for IP Western blots, anti-mouse horse radish peroxidase-conjugated secondary antibodies (Millipore, AP187P) and anti-rabbit horse radish peroxidase-conjugated secondary antibodies (Millipore, 12-348) for regular Western blots. All primary antibodies were used at 1:5000 dilution, except anti- β-Actin, which was diluted 1:50,000 for Western blot analyses. The secondary antibodies for IP were diluted 1:5000. The secondary antibodies for Western blots were diluted 1:10,000.

### Primers and siRNA

Pink1 sgRNA sequences: 5’-CACCGGTGATGTCCCTGCATGGAGCTGG-3’and 5’-AAACCCAGCTCCATGCAGGGACATCACC-3’; Pink1 Hom1 primers: 5’-TCCCCGACCTGCAGCCCAGCTCATCTCCTGAGAGCAGATCTG-3’ and 5’-CCGGAACCTCCTCCGCTCCCCAGGGCTGCCCTCCATGAGCA-3’; Pink1 Hom2 primers: 5’-AGTTCTTCTGATTCGAACATCGAACATGGCATCCTCTGTGTC-3’ and 5’-TGGAGAGGACTTTCCAAGCCTTTACTGCATGTTGACGCT-3’; siRNAs for human Fbxo7 were purchased from Santa Cruz Biotechnology (sc-75010).

### Constructs

Fbxo7 was cloned into pCI-neo with 2xMyc at its C-terminus. The *Myc-Bag2* construct was a gift from Dr. Suneil K. Kalia. *Pink1-V5* was a gift from Dr. Mark Cookson. *Pink1-Flag* was inserted into the pAdTrack vector by using NotI and HindIII. pFETCh_donor (EMMM0021) was a gift from Eric Mendenhall and Richard M. Myers (Addgene plasmid #63934; http://n2t.net/addgene:63934; RRID: Addgene_63934) [43]. pSpCas9(BB)-2A-GFP (PX458) was a gift from Feng Zhang (Addgene plasmid # 48138; http://n2t.net/addgene:48138; RRID: Addgene_48138) [44].

### Cell culture

HEK293A, SH-SY5Y and HeLa cells were cultured with 10% fetal bovine serum (ThermoFisher, 10438026) in Dulbecco's modified Eagle's medium (ThermoFisher, 11965092).

### Determination of Pink1 levels and ubiquitination

The assays were carried out as previously described [26].

### Generation of Pink1 KI cell line

The KI HEK 293A cell were generated as previously described [43, 44]. Briefly, Pink1 homologous arms were amplified by PCR using HEK 293A genomic DNA as template and assembled to the donor plasmid pFETCh_donor with Gibson Assembly (New England Biolabs, E2621) [43]. CRSPR sgRNA of Pink1 were cloned into pSpCas9(BB)-2A-GFP (PX458) [44]. Two recombinant plasmids were co-transfected into HEK 293A cells using Lipofectamine 2000 (ThermoFisher, 1168027). 24 hours after transfection, 500 μM G418 (Promega, V7983) was applied for selection of neomycin resistant cells. 2-3 weeks after G418 selection, the single colony was isolated and amplified. The amplified single colony carrying 3XFlag-P2A-neomycin cassette at Pink1 locus was confirmed by DNA sequencing and Western blotting.

### Statistical analysis

Statistical significance was determined via Prism 8.0. All data presented as mean ± SD.
